# Growing Up in Families with Parenting Stress and Conflict: Longitudinal Psychosocial Risk Patterns, Behavioral Problems and the Moderating Role of the Home Learning Environment

**DOI:** 10.3390/children13020276

**Published:** 2026-02-17

**Authors:** Susanne M. Ulrich, Anja Linberg, Sabine Düval, Susanne Kuger

**Affiliations:** 1 German Youth Institute, Department of Social Monitoring and Methodology, Nockherstr. 2, 81541 Munich, Germany; 2 Faculty of Psychology and Educational Sciences, Ludwig Maximilian University of Munich, Leopoldstr. 13, 80802 Munich, Germany

**Keywords:** risk inventory, home learning environment, child health, early childhood intervention, health service use, parenting stress, latent class analysis

## Abstract

**Highlights:**

**What are the main findings?**

- The assessed risk inventory from early childhood intervention can be used to identify psychosocial risk patterns and child development problems: children in parenting-stress/conflict-burdened and multiple-burdened families showed significantly higher levels of behavioral problems than children in low- or only economically burdened families.
- Home learning activities generally act as protective factors, but in families with high parenting stress and conflict, increased educational activity and use of universal social/educational services were associated with higher behavioral problems, suggesting that emotional and relational quality of interactions may outweigh quantity.

**What are the implications of the main findings?**

- Early identification of families experiencing parenting stress and conflict is crucial, as typical protective factors (e.g., home learning activities) may not mitigate risks and could indicate underlying relational strain.
- Universal social and educational services remain important access points for tailored support. Programs should focus not only on encouraging activity participation but also on enhancing the emotional quality, predictability, and regulation of parent–child interactions, particularly for families where interaction dynamics may otherwise undermine the benefits of these activities.

**Abstract:**

**Background/Objectives**: Assessing psychosocial burden in families can help identify those at risk and prevent negative effects on children’s well-being. This study (1) describes the longitudinal stability of psychosocial risk patterns; (2) examines group differences in the home learning environment as protective factors and in child behavior problems as an outcome; and (3) tests the moderating role of home learning activities on child behavior problems. We further explore associations with the use of institutional childcare. **Methods**: Data from 1459 children aged 0–6 years from the representative longitudinal study AID:A 2019 were analyzed across two time points (T1: 2019, T2: 2023). We tested differences in children’s behavioral problems according to risk patterns, home learning environment, and control variables, including institutional care and support service use. **Results**: The shares of families categorized as low-burdened, economically burdened, parenting-stress-and-conflict-burdened and multiple-burdened remained stable over time, even though individual stability was only moderate. Children in families with parenting stress and conflict as well as those from multiple-burdened families more frequently displayed behavioral problems at T2 than other groups. Educational activity was a protective factor for behavioral problems for most groups, but was a risk factor in conflict-and-stress-burdened families. Similar results were found for the use of universal social/educational prevention services. **Conclusions**: For most families, a better home learning environment appears to act as a buffer against the effect of risk group membership on children’s emotional well-being. However, in families marked by stress and conflict, the frequency of time together might not be beneficial—possibly because the quality of interactions matters more than the quantity. Universal social and educational services might be a place to address these families and develop targeted support.

## 1. Introduction

### 1.1. Families with Parenting Stress and Family Conflicts as a Risk Group

Psychosocial burden can complicate the transition to and early phase of family life and may have negative consequences for early childhood development and child behavior, as well as in later years. However, the question of which families should be considered “at risk” is by no means trivial—especially for families that do not fit traditional risk patterns (for example, families living in poverty). One research gap comprises families experiencing heightened parental stress and elevated conflict potential. When parents experience high levels of stress or recurrent conflict, children often are the silent recipients of the accompanying tension. Although prevalence figures in the literature vary—from approximately 12% [1] of mothers feeling continuously strained by their family responsibilities in one study to around 30% [2] in another—there is reason to believe that parenting stress is widespread. Elevated parenting stress has been linked to children’s emotional and behavioral problems [3] and was found to be transmitted directly into children’s negative temperament [4]. Even in families without socioeconomic risk factors, perfectionistic expectations and constant self-evaluation by parents can create a family climate marked by pressure and emotional unavailability. In this context, the concept of parental burnout is increasingly used in research [5,6]. It refers to a state of parental exhaustion, emotional distancing, and strong doubts about one’s own parenting competence, where the demands of parenting exceed available resources over an extended period. A recent study revealed that parenting self-efficacy mediated the correlation between parental stress and parental burnout [7].

Importantly, what parents do at home with their children may function as a moderating factor: parental behaviors and interactions can buffer against or amplify the effects of stress-related risk factors. For parenting behavior, this mechanism is relatively well studied. Findings indicate that parents suffering from parenting stress and conflict tend to show a harsh and overreactive parenting style [8]. When, in addition to stress experienced in the parental role, there is underlying anger and disagreement between partners, this negative family climate may be transmitted to the child [9]. In contrast, other facets of what parents do with their children at home—particularly shared activities that form the home learning environment (HLE)—have been examined less thoroughly. The HLE is typically discussed in relation to socioeconomic risk factors such as poverty or low parental education and is positively associated with a range of developmental outcomes. However, it remains unclear whether and to what extent the HLE can mitigate or exacerbate the developmental risks associated with parenting stress and family conflicts. Current research findings indicate that children’s processing of social information is influenced by their parents’ sensitivity. Children of highly sensitive parents show altered gaze patterns when viewing emotional facial expressions such as those of anger or joy, which suggests a possible avoidance of intense stimuli [10]. Similarly, high parental stress and family conflict may cause adults to react more quickly and in a less regulated manner to emotional stimuli, thereby influencing children’s stimulus processing and emotion regulation. Under these conditions, shared home learning activities may be experienced not as supportive, but rather as tense and inconsistent, which can be associated with an increased risk of behavioral problems in children.

While parental behaviors at home can buffer against or intensify the effects of psychosocial burden, children’s everyday environments outside the family also play an important role in shaping developmental trajectories. Early childhood education and care institutions—and later, schools—offer educational and social experiences that can support healthy development, but they can likewise become sources of stress. For young children, childcare centers may serve as key sites for early detection of psychosocial burdens and as low-threshold access points to preventive support structures [11,12]. In the context of integrated municipal strategies such as prevention chains, regular participation in childcare indicates not only successful outreach but also the extent to which families are connected to local support systems [13].

As children grow older, school environments add another layer. While they provide learning opportunities and social integration, academic pressure, behavioral expectations, and insufficient support can contribute to stress for both children and parents. Families facing higher parenting stress and familial conflicts may encounter particular challenges in navigating these institutional settings, potentially limiting their engagement with available preventive or supportive services. However, there is less research evidence on these associations.

At the same time, access to and use of childcare, school-based support, and other preventive services are not evenly distributed. Structural disadvantages—such as poverty [14], low education [15], or single parenthood [16]—shape these patterns. Families experiencing elevated parenting stress and conflict without classic socioeconomic risk factors tend to use universal medical and social/educational services to the same extent as low-burdened families, but do not use selective services to the same extent as socioeconomically burdened and multiple-burdened families [8]. This difference is central for understanding the mechanisms through which psychosocial burden affects child development. Against this backdrop, the degree of institutional embeddedness—reflected in children’s participation in childcare and school as well as families’ use of preventive services—represents an important contextual factor in our analyses.

### 1.2. Research Gaps

Despite growing evidence on the negative impact of parenting stress and family conflicts on child development, several gaps remain. First, it is still unclear how multiple risk factors, such as poverty, parental conflicts, and elevated parenting stress, are longitudinally associated with child outcomes. A recent study found that children of parents experiencing parental burnout had significantly higher rates of social anxiety, particularly when parents displayed permissive or inconsistent parenting [17]. However, the association of parenting stress and conflicts with child development difficulties is still relatively under-researched in terms of the longitudinal effects of being exposed to stress and conflict in the early years.

Second, families with high parenting stress and conflict represent a less-studied risk group compared to economically underprivileged groups. Moreover, risk patterns are rarely compared with respect to child outcomes. It remains largely unknown whether children from these families show more pronounced behavioral problems and how such differences develop over time.

Third, the home learning environment (HLE), including shared parent–child activities, has been identified as a potential protective factor. However, it is unclear whether and to what extent the HLE can moderate the impact of parenting stress and conflict on child behavior, potentially buffering or amplifying the effects of these risk factors. While prior research has examined HLE primarily in socioeconomically disadvantaged families, its role in families experiencing high stress and conflict remains underexplored.

Finally, while some interventions show promise in reducing parenting stress and conflict, it remains uncertain how such families can be reached early through preventive measures and supported in ways that strengthen parental resources, promote positive home environments, and ultimately reduce the risk of behavioral problems in children. For example, childcare centers have repeatedly been discussed as a low-threshold opportunity to strengthen and relieve families. While there is evidence that tailored programs can help reduce parenting stress [18,19], the question arises of how such families can be reached early through preventive measures in order to strengthen parental resources and counteract elevated parental stress and even chronic exhaustion. Addressing these gaps is essential for informing target-group-specific prevention and intervention strategies.

### 1.3. Study Aims

To address these gaps in the research, we examine whether children from families with high parenting stress and familial conflict potential exhibit and develop behavioral problems more frequently than children from other risk groups. This is one of the first studies to integrate HLE as a potential moderating factor in children’s behavioral problems. To this end, we use a longitudinal data set, examining data from the 2019 and 2023 waves of the AID:A study (Aufwachsen in Deutschland—Growing up in Germany) [20,21]. In the following analyses, we concentrate on children aged 0–6 years in 2019 and their early phase of development until four years later, when children are in transition to school.

This study (1) describes the stability of family risk patterns longitudinally and (2) examines group differences according to HLE as a protective factor and behavior problems as child health outcomes before (3) analyzing home learning activity as a moderating influence on child behavior problems in families with parenting stress and conflict potential in comparison to other groups. (4) Furthermore, we explore associations with the use of institutional childcare and support services.

## 2. Materials and Methods

### 2.1. Study Design

This study uses data from the population survey AID:A, in which children, adolescents, young adults, and parents of minors are asked about their living conditions and everyday experiences, and which draws on data from two points in time (T1: 2019, T2: 2023). AID:A 2019 included approximately 14,277 target individuals aged 0 to 32 from 6355 households, who were selected at random from the population registers of various sample points throughout Germany. Once the target persons had given their consent to participate in the study (in the case of minors, their primary caregivers), all other persons living in the household were to be included in the study. Thus, 9904 parents of the underage respondents also participated [22]. They answered the quantitative questionnaire in a personal interview. All participants received a small monetary incentive after participation (usually €10 each). In 2023, all participants with panel consent were again invited to take part in a replication of the 2019 study. In addition, a refreshment sample of 6500 target persons (age 0 to 32 years) improved representativeness. Finally, 12,677 target persons and 8700 parents were included in AID:A 2023 [23]. AID:A 2019 and 2023 were funded by the German Youth Institute. The study was accompanied by a scientific advisory board ensuring adherence to methodical and ethical standards. AID:A 2023 has received a positive ethical review from the Ethics Committee of the German Youth Institute (No. 2023/001). The data collection procedure was coordinated with the data protection officers of the German Youth Institute and the field institute infas (https://www.infas.de/?lang=en, accessed on 12 February 2026).

### 2.2. Instruments

#### 2.2.1. Risk Inventory from AID:A 2019 and AID:A 2023

For the following analyses, a risk inventory of 18 familial risk factors from AID:A 2019 was used. Using this inventory, we identified four distinct familial risk patterns reflecting different constellations of co-occurring risks for children aged 0–6 years. The risk inventory was originally developed for a German program in early childhood intervention with children aged 0–3 years; it covers risk factors chosen on two systematic reviews for parenting difficulties, child development problems, and child maltreatment [24,25,26]. The risk inventory covers several dimensions: the living conditions included poverty, cramped living conditions, single parent status, partner conflicts, poor social support, and low maternal education. Parental coping capabilities in response to childcare demands contained low parenting self-efficacy, anger in parenting, and poor mental health well-being. The attitude towards the child was measured by negative family climate. The childcare and parenting requirements consisted of negative emotionality towards the child for 0–3-year-old children and emotional problems for 4–6-year-old children, as well as the child’s impairment. The maladaptive parent–child dynamics included bonding problems, harsh punishment, and slapping or insulting. As far as possible, identical risk factors were used in AID:A 2023. However, some adjustments were necessary because assessment of parenting (bonding problems, slapping and insulting) switched from the child-related assessment collected from both parents in 2019 to a general score for all children in the household provided by one parent only. Therefore, the parent answer was used to assess these parenting dimensions for all children in the household. Details on the risk inventory can be found in the Supplemental Material (Table S1).

#### 2.2.2. Behavior Problems

Behavioral problems were assessed in AID:A 2023 using the Strengths and Difficulties Questionnaire (SDQ), a brief screening tool for children’s emotional and behavioral difficulties [27]. In the present study, parents rated their child’s behavior on a 3-point Likert scale (0 = “not true”, 1 = “somewhat true”, 2 = “certainly true”), for example, “Often fights with other children or bullies them”. The SDQ subscales—emotional symptoms, conduct problems, hyperactivity/inattention, and peer problems—were used to capture overall behavioral difficulties. Each subscale comprises five items, allowing the total behavioral problems score to range from 0 to 40. We then dichotomized the SDQ total score by combining the borderline range and the range giving cause for concern, before contrasting them with the normal range.

#### 2.2.3. Home Learning Activities

Home learning activities were assessed through a variety of shared parent–child activities. Following [28], these activities were categorized into educational, leisure, and digital or screen-based media activities. Educational activities included practices such as reading stories together or visiting the library [29]. Leisure activities encompassed painting, crafting, doing sports together, making music, or singing. Digital and screen-based media activities included watching videos, playing computer or mobile games, and jointly exploring information online. The frequency of each activity was reported by the parent who was considered most knowledgeable about the child’s everyday behavior, using a 6-point Likert scale (1 = never, 6 = daily). The items of each subscale were then summarized and divided by the number of items to calculate the average Likert score.

#### 2.2.4. Institutional Childcare, School Attendance, and Support Services

Parents were asked whether their child currently attends a daycare center, kindergarten, family daycare, or another form of institutional care at T1, as well as whether the child is enrolled in school at T2. In addition, parents were directly asked to indicate (yes/no) the use of various medical and social/educational and targeted services for each child. The questions on the use of support services for mothers were developed based on the insights from the Children in Germany—KiD 0–3 2015 study [15] and National Centre for Early Intervention surveys of municipalities responsible for early childhood intervention [30], funded by the Federal Ministry for Families, Senior Citizens, Women and Youth, and were expanded to cover a broader age range. 

To analyze differences between risk inventory groups, we categorized services into three subtypes [31] according to the broadness of their target group and the degree of risk, which is common in early childhood intervention [32]: universal medical services (prenatal classes, regular midwifery care after birth, postnatal exercises, and other maternal health services); universal social/educational services (parent–child groups, family or district center programs, parenting courses, telephone or online advice/counseling, exercise programs, and early childhood music education); selective services (pregnancy counseling, child guidance centers, advice on custody and contact issues, specialized counseling such as for excessive crying, and early childhood intervention such as home-visiting programs by family nurses); and services indicated by the child and youth welfare system (socio-pedagogical family midwives, or counseling provided by the youth welfare office). Utilization of a service category was coded as “used” if at least one program or service within that category was reported.

### 2.3. Analyses

#### 2.3.1. Preliminary Analyses

Latent class analysis was used to identify patterns of co-occurring psychosocial risk characteristics based on 18 indicators for parenting difficulties, developmental problems, or child welfare risks [25,26]. The AID:A 2019 study provided the basis for the data. Missing data were handled using full information maximum likelihood. Model selection considered several fit indices, with the four-class solution showing the most favorable balance of fit and parsimony according to the BIC. Details on the analyses are described elsewhere [33]. Latent class membership was used as a grouping variable for descriptive summaries and exploratory regression analyses conducted outside the latent class model. The four groups are a multiple-burdened group (4.7%), a low-burdened group (62.1%), and two medium risk groups: a group with a higher socioeconomic burden (15.7%) and a group with a higher level of parenting stress and conflict potential (17.6%). The group with parenting stress and conflict reported characteristics such as low maternal self-efficacy, anger in parenting, harsh parenting, and partner conflicts. In comparison, the economically disadvantaged group showed increased characteristics such as an elevated risk of poverty, living in cramped housing conditions, or low education. While the group of low-burdened families could hardly be characterized by any of these characteristics, the group of multiple-burdened families featured nearly all risk factors.

#### 2.3.2. Current Analyses

The four psychosocial risk groups were used to examine group differences in HLE activities and behavioral problems. Differences in frequencies between the risk groups were tested for statistical significance (*p* < 0.05) using a chi-square test. For statistical ANOVA post hoc tests were performed for statistical validation. We then tested differences in children’s behavioral problems according to risk group membership, HLE activities, and control variables (child’s gender, age, institutional care, school attendance) in logistic regression models, including the sets of variables stepwise. To assess whether HLE activities moderated the association between risk group membership and children’s behavioral problems, we included interaction terms between risk groups and HLE activities. For ease of interpretation, we report the interaction effects as average marginal effects (AMEs). AMEs represent the average change in the predicted probability of the outcome associated with a one-unit change in a predictor, calculated across all observations while holding all other variables at their observed values. This approach allows for a more intuitive interpretation of effect sizes than odds ratios or logit coefficients. In addition, we analyzed the association between behavior problems and the use of several support systems across risk groups and tested corresponding interaction terms. Missing data among the independent variables were generally low: missing values for the three HLE activities were below 1% among parents who responded to the child questionnaire in this subsample, and missing values for the support service variables ranged between 1.85% and 2.60%. Associations were therefore estimated using listwise deletion, in line with current recommendations for large, representative samples with only small proportions of missing data [34]. For the SDQ outcome, analyses were based on *n* = 820 observations in the longitudinal subsample. This reduced sample size does not reflect missing item responses, but results from the survey design. SDQ data in 2023 were collected only if the child had responded to at least one SDQ item in 2019. This filter was part of an exploratory procedure to test the practicability of the SDQ instrument in AID:A and is described in detail elsewhere. Since design decisions were not based on family risk characteristics, the absence (or existence) of SDQ information is independent from the dependent variable (LCA profiles) or any of its individual contributions (risk factors). All analyses were conducted using Stata 18.

## 3. Results

Table 1 presents sociodemographic characteristics of families with children aged ≤ 6 years in 2019 and aged 3–11 years in 2023. The average age of parents and children increased accordingly, while the distribution of family types remained largely stable. Most households had one or two children under 18, and educational levels showed little change across waves. The proportion of families with a migration background also remained consistent. Notably, dual-parent employment increased substantially from 66.3% to 83.4%, while the share of households below 60% of the median net equivalent income decreased slightly.

**Figure 1:**
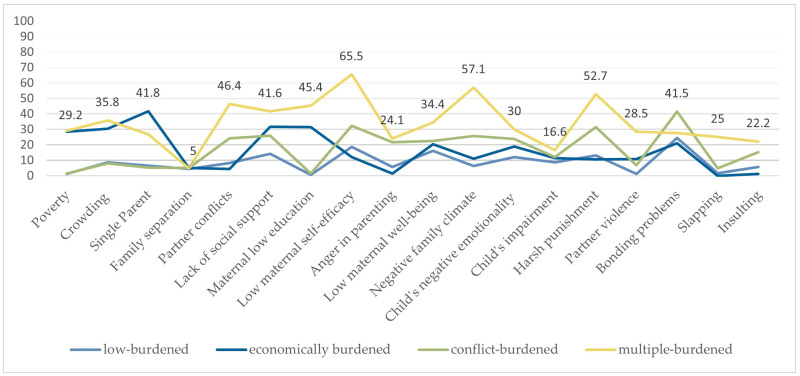
Percentage shares of psychosocial risk factors at T2 by risk group at T1, n_max_ = 1459.

Figure 1 shows the percentage shares of psychosocial risk factors at T2 by risk group at T1: low-burdened, economically burdened, stress-and-conflict-burdened, and multiple-burdened families. The values represent the proportion of children within each risk group who show the respective characteristic at T2. For example, 28.5% of children from multiple-burdened families at T1 exhibit poverty at T2.

Families with parenting stress and conflict at T1 still exhibit particularly high rates in domains directly linked to interpersonal tension, such as partner conflicts, negative family climate, harsh parenting, and partner violence at T2. In contrast, economically burdened families tend to continuously score higher only on structurally related risks (e.g., poverty, low education), while low-burdened families later on also show uniformly low levels across all domains. Multiple-burdened families show the consistently highest probabilities across almost all indicators, spanning socioeconomic strain, family instability, parental well-being, and child emotional or behavioral difficulties. Further details can be found in Table S2.

**Figure 2:**
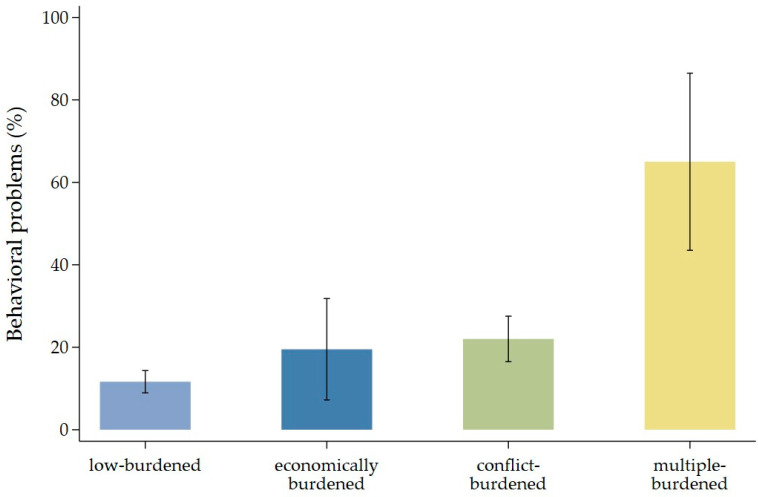
Percentage shares of behavioral problems (SDQ total) at T2 by risk group at T1, n = 820.

Figure 2 shows the mean percentage (with 95% confidence intervals) of children with behavior problems at T2 across the four risk groups. The results reveal a clear gradient: low-burdened families show the smallest share of children with behavior problems, while their percentage increases steadily across the economically and parenting-stress-and-conflict-burdened groups. Families with parenting stress and conflict display noticeably higher shares of children with behavior problems compared to the low-burdened group. The highest percentage of child behavior problems—exceeding 60%—appears among multiple-burdened families.

**Figure 3:**
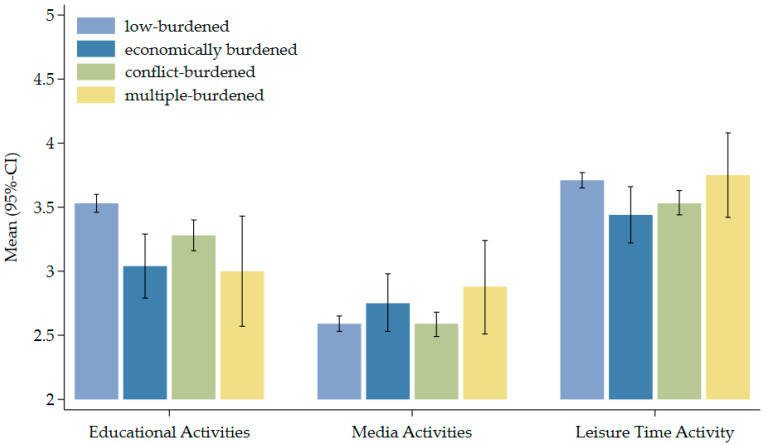
Mean levels of educational (*n* = 1104), media (*n* = 1105), and leisure-time (*n* = 1104) activity at T2 by risk group at T1. Note: Education: reading stories aloud, going to the library together; media: using video services, playing games on the computer/mobile phone, searching for things on the internet together; leisure time: painting, crafts, handicrafts, playing sports together, making music or singing songs together.

Figure 3 displays mean levels (with 95% confidence intervals) of parental involvement in the three HLE activities of education, media, and leisure time across the four risk groups. All burdened families showed significantly fewer educational activities than the low-burdened families. Regarding media activity, all groups show generally lower involvement, but economically and multiple-burdened families exhibit higher engagement compared to the other groups. However, ANOVA post hoc tests were not significant. On the right of Figure 3, the data reveal that stress-and-conflict-burdened families shared significantly fewer leisure-time activities than low-burdened families. In contrast, multiple-burdened families display levels that largely converge with the economically burdened group, though multiple-burdened families show consistently elevated variability.

Table 2 confirms the results from descriptive analyses that children experiencing parenting stress and conflict as well as multiple burdens are more likely to display behavior problems. Overall, educational activities tend to function as a protective factor (Model 3, OR = 0.7), but the interaction terms are significantly associated with worse outcomes for children in stress-and-conflict-burdened families (Model 4, OR = 1.6). Media time shows inconsistent associations, with small and mostly non-significant associations across groups, while leisure-time activities remain largely unrelated to behavioral problems. The inclusion of control variables—such as child age, childcare attendance, and school participation—adjusts the estimates modestly and does not change the overall pattern of results. However, gender is significantly associated in all models.

**Figure 4:**
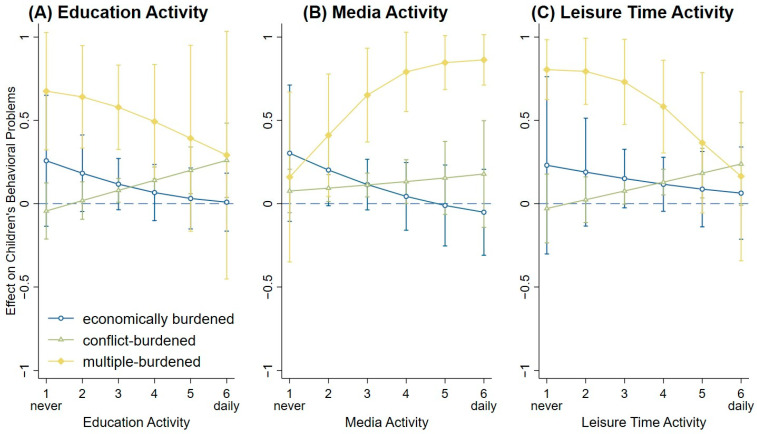
Predicted probabilities of children’s behavioral problems by risk group across levels of HLE activities (educational, media, and leisure-time activity). Note: Margin plots from logistic regression models showing average marginal effects (AMEs) for the interaction between risk groups (low-burdened [reference], economically burdened, conflict-burdened, and multiple-burdened) and each of the three home learning environment (HLE) activities. Separate logistic regression models were estimated for (**A**) educational activity, (**B**) media activity, and (**C**) leisure-time activity, each measured on a scale from 1 = never to 6 = daily. The plots display the predicted probability of children’s behavioral problems across HLE activity levels for each non-reference risk group. All predictions are adjusted for control variables.

Figure 4 displays the average marginal effects (AMEs) of shared family activities on the predicted probability of children’s behavioral problems across psychosocial risk patterns. Across the three domains—educational activity, media activity, and leisure-time activity—the AMEs for economically burdened families are relatively small and do not differ statistically significantly from those of the low-burdened reference group. The associations are comparable in magnitude to those observed in families experiencing parenting stress and conflict, where children exhibit higher predicted probabilities of behavioral problems than those in the low-burden reference group, as indicated by positive AMEs. For this group, the AMEs tend to increase slightly with more shared family time activities, although only some of these differences reached statistical significance. Children from multiple-burdened families consistently show the largest positive AMEs across all activity domains, indicating the highest predicted probabilities of behavioral problems. In two of the three domains—educational and leisure-time activities—the AMEs decrease significantly with increasing shared family time, suggesting a lower level of behavioral problems. An exception is family media activity, which is associated with significantly higher behavioral problems in multiple-burdened families. A sub-analysis of individual media items did not yield statistically significant results for this group, likely due to the small sample size. Further details can be found in Table S3.

Next to gender and age, we also included institutional care and school attendance as control variables in order to assess the role of institutions, especially for developing preventive support. Across the regression models, the use of school and institutional childcare does not play a meaningful role in explaining variation in children’s behavioral difficulties. However, the uptake of support services reveals important differences: the use of universal social and educational programs is associated with more behavioral problems, particularly among stress-and-conflict-burdened families, suggesting that shared time—both within the family and in institutional settings—may be more challenging than beneficial for this group (see Table S4). In contrast, the use of selective support services indicates that children with pronounced behavioral problems are disproportionately represented among economically and multiple-burdened families. This pattern reflects a classic prevention dilemma [35], in which those most in need of early support are concentrated in targeted programs, while universal services may not sufficiently mitigate, and may even coincide with, elevated stress and behavioral difficulties.

## 4. Discussion

In short, the results suggest that children from families experiencing parenting stress and conflict potential, as well as multiple-burdened children, showed more frequent behavioral problems after four years than children from other risk groups. Although educational activities are generally considered a protective factor, they are associated with a slightly higher likelihood of behavioral problems among children in families with parenting stress and conflict. The data tend to further show associations between family media time as well as family leisure time and behavioral problems in these children. These families used social and educational services more frequently than the other risk groups, whereby the usage was also associated with problematic child behavior. This might be a hint that spending time together within the family and also in institutional contexts does not solve the underlying tensions in this group. By contrast, although children from multiple-burdened families show the highest overall levels of behavioral problems, they appear to benefit substantially from increased family education time and family leisure time. However, more family media time in this group is associated with more pronounced behavioral problems.

### 4.1. Longitudinal Psychosocial Risk Patterns

The risk groups formed in AID:A 2019 with a latent class analysis showed similar patterns in the distribution of the risk factors four years later. Thus, the identified parenting-stress-and-conflict-burdened families had frequent partner conflicts, lack of social support, low maternal self-efficacy, harsh parenting, and bonding problems at both timepoints. Economically burdened families had a higher probability of suffering from poverty, single parenthood, or low education than other risk groups. These four patterns were already found in other studies with other age groups [24,36,37]. The results here present a certain stability of risk factors over time and therefore a transferability of the risk inventory to older age groups, not only for socioeconomic factors as seen in numerous studies (e.g., [38]), but also in parent characteristics and parent–child dynamics. However, further research on the stability and change of risk patterns is needed, to clarify how many children switch between risk profiles and which factors are decisive for a change in these patterns.

### 4.2. Behavioral Problems as Child Health Outcome

The findings show that a high level of child behavioral problems in young children is more common when cumulative risk factors are present. In addition, the risk patterns of the factors have also proven to be relevant. Children in families with parenting stress and conflict are significantly more likely to exhibit behavioral problems than children from families with primarily socioeconomic stress, with an average of around four stress factors reported in both groups. This is not surprising, given the fact that witnessing conflicts and a temporary reduction in parental care and parenting skills, as experienced by children in parents’ divorces, are important mediating mechanisms for the negative family climate [39,40]. For example, children who witness more intense or chronic parental conflicts after a separation are on average more severely affected than children who do not. However, chronic conflicts, especially in the parents’ cooperation in caregiving and parenting, also affect children even if the parents do not separate (e.g., [18]). Other findings also show that when parents experience stress in the parental role or anger and disagreement arise between partners, the negative family climate may be transmitted to the child [9].

### 4.3. Home Learning Activity as a Moderating Factor on Child Behavioral Problems in Risk Groups

While in educational research there seems to be a wide consensus that HLE generally supports children’s long-term development [41], our results add further insights. HLE differentially affects children’s development depending on family risk patterns. In economically burdened families, children seem to profit continuously from more shared activities. This is in line with other findings, in which HLE acts as a moderator for socioeconomic disparities, and where rich environments can bridge the gap in school readiness [42,43]. In contrast, the opposite is true in stress-and-conflict-burdened families. The most probable reason for this is that the quality of HLE has in the past proven to be more important than pure quantity. High intensities of “low-quality” interactions in a family seem to lead to more behavioral problems. These families may represent emotionally intense and poorly regulated environments. Research on parental emotional reactivity and emotion-processing biases suggests that heightened stress can be associated with increased emotional volatility, reduced regulatory capacity, and less predictable interaction patterns [3,44]. Shared activities in such contexts may therefore be experienced by children as emotionally demanding or overwhelming, which could accumulate over time and contribute to behavioral difficulties. As a consequence, survey studies should move from assessing only quantity indicators for HLE to including some quality indicators as well. Interestingly, HLE activities affect children in multiple-burdened families in a non-linear manner. While particularly high doses of educational and leisure-time activities exert protective effects, leading to less problematic behavior, even small doses of media activities seem to exacerbate problematic behavior tremendously. These findings may be explained by the emotional context in which media use occurs. In families experiencing multiple burdens, media activities may contribute to emotional overstimulation or occur in settings with less co-regulation and parental guidance [45], which can intensify behavioral difficulties. In contrast, structured educational and leisure activities may provide more predictable and regulated interaction patterns that buffer against stress. These results provide insights for designing targeted family training programs, suggesting that risk groups should be used to carefully monitor and shape family activities differentially, emphasizing emotionally attuned and regulated engagement rather than simply the frequency of activities.

### 4.4. The Use of Institutional Childcare and Prevention Services

Institutional care and school attendance were not significant control variables in our regression models. Increasing institutional involvement with advancing child age brings with it further risks (e.g., school stress) and opportunities (e.g., friendships and peer groups), which should be taken into account in future studies when considering risk groups. In order to further develop appropriate prevention measures, it is important to find out whether increased parenting stress is also associated with greater use of support services. Evidence was found that families with increased parenting stress and family conflict often use universal services such as parent–child groups, but are less likely than economically disadvantaged families to seek help from targeted services (e.g., parenting advice at child guidance centers) [8]. It is possible that families from this group may conceal parenting stress, mental health issues, and familial conflicts, so that the need for help is less visible to professionals and more broadly. A supposedly good social situation with respect to financial resources, income, work, and communication style can contribute to underestimating the burden. The fear of losing control and the fear of being stigmatized as a “bad parent” can be barriers to utilizing support services [46]. Accordingly, such beliefs, which may also be inherent in one’s personality [47], can impede the use of support services despite increased stress. In sum, a research gap still exists when it comes to understanding the characteristics of these families, e.g., their work–life balance and socioeconomic situation.

One strength of this study is the large, nationally representative, and longitudinal sample that allows us—partly—to go beyond cross-sectional associations and to analyze risk and developmental patterns. In addition, it is one of the few studies that records the use of various family support services, allowing for some considerations regarding the need for support and prevention. Of course, the study also has some limitations. The data are based on self-reported information from parents, which may be distorted by social desirability, but this allows us to obtain detailed information on a variety of family characteristics that cannot be collected economically efficiently by other means. From a methodological perspective, analyses comparing classes on external variables should be considered exploratory, as the group assignments are based on probabilities. As these analyses do not adjust for classification uncertainty—and given the limited classification precision indicated by the entropy of the class solution—findings involving class membership should be interpreted cautiously. Future studies should apply fully model-based approaches (e.g., BCH or multistep methods) to account for classification error.

## 5. Conclusions

For most families, increased family educational and leisure time help protect children’s emotional well-being. However, in families marked by parenting stress and conflict potential, this same type of time together might not be beneficial—possibly because the quality of interactions matters more than the quantity. Additionally, family media use should be closely monitored, as it appears particularly relevant for children from multiple-burdened families, where higher family media time is associated with increased behavioral problems. Universal social and educational services might provide an arena in which to address these families and develop targeted support. To this end, professionals must be encouraged to proactively recognize needs for assistance that are difficult to identify.

## Figures and Tables

**Table 1:** Sociodemographic factors for sample with children ≤ 6 years at AID:A 2019 and 3–11 at AID:A 2023, n_max_ = 1459.

Sociodemographics		AIDA 2019 M (SD)|%	AIDA 2023 M (SD)|%
Mean age of biological mother		35.2 (5.00)	39.13 (5.00)
Mean age of biological father		38.36 (5.86)	42.30 (5.87)
Mean age of child		3.20 (1.93)	7.18 (1.96)
	nuclear family	88.46	86.15
	single parents	6.11	9.18
	stepfather family	5.00	4.66
Number of children < 18 per household	0	13.43	14.82
	1	44.85	49.42
	2	30.46	28.34
	3 and more	11.26	7.36
Highest education level in household	low	5.30	3.79
	medium	50.14	52.05
	high	44.56	44.16
Migration background	child or both parents	11.61	11.59
	one parent	12.5	12.48
	none	75.89	75.93
Employment	none	3.18	1.9
	only father	29.2	12.86
	only mother	1.31	1.83
	both parents	66.3	83.42
Beyond 60% of median net equivalent income	yes	13.25	10.83
Institutional care of child	yes	71.24	16.24
School attendance	yes	9.80	50.51

**Table 2:** Logistic regression models (odds ratios and standard errors) of behavior problems according to risk groups.

		(1)	(2)	(3)	(4)	(5)	(6)	(7)	(8)	(9)	(10)
**Outcome: Children’s Behavioral Problems**		**Conflict Group**	**Education**	**Moderator: Education**	**Moderator: Education + Controls**	**Media**	**Moderator: Media**	**Moderator: Media + Controls**	**Leisure Time**	**Moderator: Leisure Time**	**Moderator: Leisure + Controls**
Risk group	Economically	1.84 (0.77)	1.70 (0.71)	2.55 (3.05)	4.52 (5.74)	1.76 (0.74)	8.95 (12.05)	9.96 (14.18)	1.81 (0.75)	2.39 (3.82)	3.69 (6.24)
(Ref.: Low-burdened)	Conflict	2.14 *** (0.45)	2.04 *** (0.44)	0.497 (0.35)	0.45 (0.40)	2.14 *** (0.45)	1.85 (1.30)	1.73 (1.38)	2.10 *** (0.44)	1.12 (1.05)	0.54 (0.59)
	Multiple	14.09 *** (6.87)	13.92 *** (6.81)	37.10 (76.02)	38.90 (81.03)	13.65 *** (6.68)	0.66 (1.25)	1.05 (2.29)	14.87 *** (7.31)	489.8 * (1430.45)	526.0 * (1625.91)
**Education Activity**			0.83 (0.08)	0.73 * (0.09)	0.78 (0.13)						
Interaction	Economically			0.85 (0.34)	0.80 (0.33)						
Ref.: (Low-burdened	Conflict			1.55 * (0.32)	1.59 (0.39)						
X Education Activity)	Multiple			0.76 (0.43)	0.78 (0.47)						
**Age in Years**					0.95 (0.11)			1.00 (0.11)			0.98 (0.11)
**Girl (Ref.: Boy)**					0.49 ** (0.11)			0.49 ** (0.11)			0.49 ** (0.11)
**Institutional Childcare (Ref.: No)**					1.44 (0.56)			1.45 (0.56)			1.49 (0.58)
**School Attendance (Ref.: No)**					1.15 (0.46)			1.04 (0.41)			1.04 (0.41)
**Media Activity**						1.18 (0.14)	1.15 (0.19)	1.05 (0.20)			
Interaction	Economically						0.56 (0.27)	0.62 (0.31)			
(Ref.: Low-burdened X	Conflict						1.06 (0.26)	1.10 (0.31)			
Media Activity)	Multiple						3.11 (2.20)	2.99 (2.50)			
**Leisure-Time Activity**									0.85 (0.10)	0.84 (0.14)	0.82 (0.17)
Interaction	Economically									0.92 (0.42)	0.91 (0.43)
(Ref.: Low-burdened X	Conflict									1.20 (0.31)	1.50 (0.45)
Leisure-Time Activity)	Multiple									0.42 (0.30)	0.45 (0.33)
	N	820	818	818	681	819	819	681	818	818	681
	F	38.21 ***	41.64 ***	47.59 ***	54.31 ***	40.20 ***	45.84 ***	52.45 ***	0.0881 ***	42.61 ***	53.01 ***
	R^2^	0.05	0.06	0.07	0.09	0.06	0.06	0.09	0.06	0.06	0.09

Note: * *p* < 0.05, ** *p* < 0.01, *** *p* < 0.01

## Data Availability

Data are available under https://surveys.dji.de/index.php?m=msw,0&sID=118, accessed on 12 February 2026.

## Supplementary Materials

The following supporting information can be downloaded at: https://www.mdpi.com/article/10.3390/children13020276/s1, Table S1: Risk factors on family situation, parent and child characteristics and parenting, weighted n_max_ = 3744 AIDA 2019 [48,49,50,51,52,53,54,55,56,57,58,59,60,61]; Table S2: Percentage shares of psychosocial risk factors at T2 by risk group at T1; Table S3: Average marginal effects for child behavior problems and home learning environment moderators; Table S4: Odds ratios for child behavior problems according to risk groups and preventive service use.

## Abbreviations

The following abbreviations are used in this manuscript:

AID:A	Aufwachsen in Deutschland—Growing Up in Germany
AMEs	Average Marginal Effects
HLE	Home Learning Environment
SDQ	Strengths and Difficulties Questionnaire
