# Supplementary material for: Growing Up in Families with Parenting Stress and Conflict: Longitudinal Psychosocial Risk Patterns, Behavioral Problems and the Moderating Role of the Home Learning Environment
Source: Children (Basel). 2026 Feb 17;13(2):276. doi: 10.3390/children13020276 (PMC12939705; doi:10.3390/children13020276)
Supplement: Supplementary file 1 [file children-13-00276-s001.zip › children-4084424-supplementary.pdf]

## Supplement Material

**Table S1.** Risk factors on family situation, parent and child characteristics and parenting, weighted $n_{max}$=3744 AIDA 2019.

| Risk factor T1 | Definition | Scale | Cut-off | α |
|---|---|---|---|---|
| **Living conditions** | | | | |
| Poverty | Receivement of social welfare in household | 0;1 | 1 | |
| Crowding | Number of children in household/Number of children's room < 1.5 based on US Definition of crowded living conditions [48] | 0;6 | >=1.5 | |
| Single Parent | One parent with at least one child in household with or without further persons in household [49] | | | |
| Post-separation family | At least one child in the household is not the biological child of both parents living in the household [49] | | | |
| Partner conflicts | Frequent loud quarrels according to at least one parent [50] | 1;6 | <4 | |
| Partner violence | At least seldon violent assaults asccording to at least one parent [50] | 1;6 | <6 | |
| Lack of social support | Poor social support according to at least one parent in household [51] | 3;15 | <=8 | 0.50 |
| Maternal low education | At least a secondary school leaving certificate and no vocational qualification of the mother [52] | | | |
| **Parental characteristics for coping capabilities in response to child care demands** | | | | |
| Low maternal self-efficacy | Feeling overwhelmed according to mother [5] | 1;6 | >=4 | |
| Anger in parenting | Maternal agreement on "I get angry quickly when my child doesn't do what I say" [53] | 1;6 | <4 | |
| Maternal low mental well-being | Poor wellbeing according to WHO-5 [54,55] according to mother | 0;100 | <50 | 0.79 |
| **Attitute towards the child** | | | | |
| Negative family climate | 4 items on family cohesion (enjoy being with family [reversed], friction, being able to talk about everything [reversed], strong cohesion [reversed]) according to at least one parent [56] | 4;24 | >=11 | 0.48 |
| **Child care and parenting requirements** | | | | |
| Child's negative emotionality | 3 items on negative emotionality for 0-3 year olds (excitable, difficult to comfort, happy/satisfied [reversed]) [57] and SDQ-Subscale [27] with anbnormal values on emotional problems according to the respondent parent for children >=4 years | 0:15<br>1;15 | >=9<br>>4 | 0.53 |
| Child's impairment (e.g. disability, chronic illness, allergy) | Disability or impairment; this refers to long-term physical, cognitive, mental, emotional or health-related conditions that can lead to restrictions in everyday life according to the person providing information in the household [58] | 0;1 | 1 | |
| **Maladaptive parent-child-dynamics** | | | | |
| Bonding problems | lack of empathy (e.g. difficult to understand the child's feelings, get worried if child is upset or distresses, concerns me when misfortunes happen to him/her, try to see things from his eyes) according to at least one parent [59,60] | 0;4 | >=3 | 0.05 |
| Harsh punishment | Parental agreement on „I punish my child harshly, even for small things“ according to at least one parent [61] | 1;6 | <5 | |
| Slapping (child maltreatment) | Parental agreement on „If my child is naughty or behaves inappropriately, I give him a slap or a slap in the face“ according to at least one parent [61] | 1;6 | <5 | |
| Insulting | Parental agreement on „If my child is naughty or behaves inappropriately, I say mean or hurtful things or call them names“ according to at least one parent [61] | 1;6 | <5 | |

**Table S2.** Percentage shares of psychosocial risk factors at T2 by risk group at T1

| | Low-burdened | Economically burdened | Conflict-burdened | Multiple-burdened |
|---|---|---|---|---|
| Poverty | 1.14 | 28.50 | 1.38 | 29.20 |
| Crowding | 8.76 | 30.50 | 7.95 | 35.80 |
| Single Parent | 6.54 | 41.80 | 5.27 | 26.80 |
| Separation family | 4.46 | 5.10 | 5.00 | 4.87 |
| Partner conflicts | 8.23 | 4.34 | 24.30 | 46.40 |
| Lack of social support | 14.20 | 31.60 | 25.90 | 41.60 |
| Maternal low education | 0.67 | 31.50 | 1.52 | 45.40 |
| Low maternal self-efficacy | 18.60 | 12.10 | 32.40 | 65.50 |
| Anger in parenting | 5.69 | 1.51 | 21.60 | 24.10 |
| Low maternal well-being | 16.20 | 20.30 | 22.60 | 34.40 |
| Negative family climate | 6.26 | 11.10 | 25.70 | 57.10 |
| Child's negative emotionality | 12.10 | 18.90 | 23.70 | 30.00 |
| Child's impairment | 8.59 | 11.50 | 12.00 | 16.60 |
| Harsh punishment | 13.10 | 10.50 | 31.40 | 52.70 |
| Partner violence | 1.20 | 10.80 | 6.68 | 28.50 |
| Bonding problems | 24.40 | 21.00 | 41.50 | 27.70 |
| Slapping | 1.70 | 0.00 | 4.81 | 25.00 |
| Insulting | 5.68 | 1.28 | 15.20 | 22.20 |

**Table S3.** Average marginal effects for child behavior problems and home learning environment moderators

| | (1) Conflict Group | (2) Moderator: Education + Controls | (3) Moderator: Media + Controls | (4) Moderator: Leisure Time + Controls |
|---|---|---|---|---|
| Economically burdened | 0.079 | | | |
| Conflict-burdened | $0.10^{***}$ | | | |
| Multiple-burdened (Reference: Low-burdened) | $0.53^{***}$ | | | |
| Economically burdened X Education Activity | | | | |
| Never | | 0.26 | 0.30 | 0.23 |
| Seldom | | 0.18 | 0.20 | 0.19 |
| 1-2 times per month | | 0.12 | 0.11 | 0.15 |
| 1-2 times per week | | 0.07 | 0.04 | 0.12 |
| Several times per week | | 0.031 | -0.01 | 0.09 |
| Daily | | 0.01 | -0.05 | 0.06 |
| Conflict-burdened X Media Activity | | | | |
| Never | | -0.04 | 0.08 | -0.03 |
| Seldom | | 0.02 | $0.09^{*}$ | 0.02 |
| 1-2 times per month | | $0.08^{*}$ | $0.11^{**}$ | 0.08 |
| 1-2 times per week | | $0.14^{**}$ | $0.13^{*}$ | $0.13^{**}$ |
| Several times per week | | $0.20^{**}$ | 0.15 | $0.18^{*}$ |
| Daily | | $0.26^{*}$ | 0.18 | 0.24 |
| Multiple-burdened X Leisure Time Activity | | | | |
| Never | | $0.68^{***}$ | 0.16 | $0.81^{***}$ |
| Seldom | | $0.64^{***}$ | $0.41^{*}$ | $0.79^{***}$ |
| 1-2 times per month | | $0.58^{***}$ | $0.65^{***}$ | $0.73^{***}$ |
| 1-2 times per week | | $0.49^{**}$ | $0.79^{***}$ | $0.58^{***}$ |
| Several times per week | | 0.39 | $0.85^{***}$ | 0.37 |
| Daily | | 0.29 | $0.86^{***}$ | 0.16 |
| N | 820 | 681 | 681 | 681 |
| F | 38.21 | $54.31^{***}$ | $52.45^{***}$ | $53.01^{***}$ |
| $R^2$ | 0.05 | 0.09 | 0.09 | 0.09 |

$^{*}$ $p < 0.05$, $^{**}$ $p < 0.01$, $^{***}$ $p < 0.001$

**Table S4.** Odds ratios for child behavior problems according to risk groups and preventive service use

| | | a1 | a2 | a3 | a4 | a5 | a6 |
|---|---|---|---|---|---|---|---|
| | | Conflict group | School Attendance | Social services | Medical services | Selective services | Indicated services |
| Risk groups | Economically burdened | 1.9 | 3.31+ | 0.79 | 0.45 | 2.80+ | 1.6 |
| | Conflict-burdened | 4.00* | 2.68* | 0.51 | 0.73 | 1.74* | 2.40*** |
| | Multiple-burdened | 12.60*** | 11.91*** | 11.81* | 13.33*** | 14.72*** | 11.70*** |
| Inst. Childcare (Ref.: No) | | 2.11 | | | | | |
| Risk groups X institutional care | Economically burdened | 1.4 | | | | | |
| | Conflict-burdened | 0.51 | | | | | |
| | Multiple-burdened | 1 | | | | | |
| School Attendance (Ref.: No) | | | 1.44 | | | | |
| Interaction School Attendance | Economically burdened | | 0.44 | | | | |
| X Risk groups | Conflict-burdened | | 0.74 | | | | |
| (Ref.: Low-burdened X No service use) | Multiple-burdened | | 2.03 | | | | |
| Social/ educational services | | | | .43** | | | |
| Interaction social/educational services | Economically burdened | | | 3.19 | | | |
| X Risk groups | Conflict-burdened | | | 5.55** | | | |
| | Multiple-burdened | | | 1.17 | | | |
| Medical services | | | | | .29*** | | |
| Interaction Medical services | Economically burdened | | | | 5.32 | | |
| X Risk groups | Conflict-burdened | | | | 3.61+ | | |
| (Ref.: Low-burdened X No service use) | Multiple-burdened | | | | 1 | | |
| Selective services | | | | | | 1.36 | |
| Interaction Selective Services | Economically burdened | | | | | 0.44 | |
| X Risk groups | Conflict-burdened | | | | | 2.36+ | |
| (Ref.: Low-burdened X No service use) | Multiple-burdened | | | | | 0.70 | |
| Indicated services | | | | | | | 6.38*** |
| Interaction Indicated services | Economically burdened | | | | | | 0.38 |
| X Risk groups | Conflict-burdened | | | | | | 0.08* |
| (Ref.: Low-burdened X No service use) | Multiple-burdened | | | | | | 0.47 |
| | N | 678 | 820 | 811 | 810 | 807 | 808 |
| | F | 30.67*** | 41.09*** | 45.33*** | 41.17*** | 47.73*** | 49.90*** |
| | $R^2$ | 0.0525 | 0.0568 | 0.0644 | 0.0594 | 0.0676 | 0.0713 |

Note: + p < 0.10; * p < 0.05; ** p < .01; *** p < .001
